# Research progress of rhizosphere microorganisms in *Fritillaria* L. medicinal plants

**DOI:** 10.3389/fbioe.2022.1054757

**Published:** 2022-11-07

**Authors:** Nong Zhou, Chun-Mei Mei, Xing-Yu Zhu, Jing-Jing Zhao, Ming-Guo Ma, Wei-Dong Li

**Affiliations:** ^1^ College of Pharmacy, Engineering Center of State Ministry of Education for Standardization of Chinese Medicine Processing, Nanjing University of Chinese Medicine, Nanjing, China; ^2^ Chongqing Engineering Laboratory of Green Planting and Deep Processing of Famous-region Drug in the Three Gorges Reservoir Region, College of Biology and Food Engineering, Chongqing Three Gorges University, Chongqing, China; ^3^ Research Center of Biomass Clean Utilization, Beijing Key Laboratory of Lignocellulosic Chemistry, College of Materials Science and Technology, Beijing Forestry University, Beijing, China

**Keywords:** *Fritillaria* L., soil, rhizosphere microbes, microbiome, research progress

## Abstract

The soil’s rhizosphere is a highly active place where the exchange of substances and information occurs among plants, soils, and microorganisms. The microorganisms involved are crucial to the activities of plant growth and development, metabolism, and reproduction. *Fritillaria* L. medicinal plants are unique Chinese medicinal ingredients, but the continuous cropping obstacles formed in the artificial planting process is severely harmful to the growth and development of these medicinal plants. In this review, we summarized the current species and distribution of *Fritillaria* L. in China, and analyzed the changes in microbial diversity (mainly among bacteria and fungi) in the rhizosphere of these plants under long-term continuous cropping. The fungi showed an increasing trend in the soil rhizosphere, resulting in the transition of the soil from the high-fertility “bacterial type” to the low-fertility “fungal type” as planting years increased. Furthermore, the interaction between *Fritillaria* L. medicinal plants and the rhizosphere microorganisms was reviewed, and promising applications for the rhizosphere microbiome in the cultivation of *Fritillaria* L. medicinal plants were suggested. It is expected that this review will facilitate the in-depth understanding of rhizosphere microorganisms in the growth, accumulation of active ingredients, and disease control of *Fritillaria* L.

## Introduction

*Fritillaria* L., a valued traditional Chinese herbal medicine, contains diterpenoids, steroids, alkaloids, polysaccharides, which have broad applications in health care products, food, Chinese herbal formulas, and everyday chemical industries. This plant has the benefits of reducing phlegm, relieving coughs, reducing heat, moistening the lungs, improving blood stasis, and dispersing knots (Chen et al., 2020). It is also one of the main ingredients in cough- and phlegm-relieving Chinese patented medicines. However, the sources of *Fritillaria* medicinal plants in the wild are becoming increasingly reduced due to its small output, long resource regeneration cycle, overexploitation, and habitat destruction. In 2021, it was listed as a secondary protected plant among the national key protected wild plants (Cunningham et al., 2018). Moreover, the quantity and quality of *Fritillaria* medicinal materials on the market are inconsistent because of the wide variety of *Fritillaria* medicinal plants, the wide production area, and the lack of standardized processing and operation processes in planting and production. There are problems in concerning the medicinal and edible safety of *Fritillaria* medicinal materials, which are restricting the development of the *Fritillaria* medicinal plant industry (Chen et al., 2021). Therefore, developing artificial and standardized cultivation methods for *Fritillaria* medicinal plants is the most effective way to solve the contradiction between the current market demand for medicinal materials and its resource protection.

The rhizosphere is a unique ring-shaped zone mainly characterized by the interaction between the root system and soil microorganisms. A large number of microorganisms, such as bacteria, Actinomycetes, fungi, and soil animals, gathered around the plant root system, which is a part of the soil microenvironment that presenting special physical, chemical, and biological properties (Garcia and Kao-Kniffin, 2018; Mojicevic et al., 2019; Shao et al., 2021). As the most active component in the soil ecosystem, soil microbes mainly participate in the cycling of nutrients, such as carbon, nitrogen, phosphorus, and sulfur in the soil (Hemkemeyer 2021). Rhizosphere microbes improves the utilization rate of nutrients in the soil, thereby affecting the growth and development of plants and stress resistance, and promoting good plant health and soil quality. Therefore, it is of great significance to perform research on the rhizosphere microorganisms of medicinal plants (Mendes et al., 2013; Tian et al., 2018). However, few reports exists on the rhizosphere microorganisms of *Fritillaria* medicinal plants (Qiu, 2010; Pan et al., 2010a,Pan et al., 2010b; Mu et al., 2019a,Mu et al., 2019b,Mu et al., 2019c; Wu et al., 2021b).

This review article systematically describes the research results on the rhizosphere microorganisms associated with *Fritillaria* medicinal plants in detail. The words “*Fritillaria*,” “rhizosphere microorganisms,” and “Microbiome” were used as keywords to search the literature in the Chinese National Knowledge Infrastructure (CNKI) and Web of Science. Fifty-seven literature was searched in CNKI, and 57.9% of which was published from 2016 to 2021, six literature was searched in Web of Science. It is expected that this review will contribute to further understand the involvement of rhizosphere microorganisms in the growth of *Fritillaria* medicinal plants and the accumulation of alkaloids, which are the most important active ingredients of *Fritillaria*.

## Classification of medicinal *Fritillaria* plants in China

**FIGURE 1:**
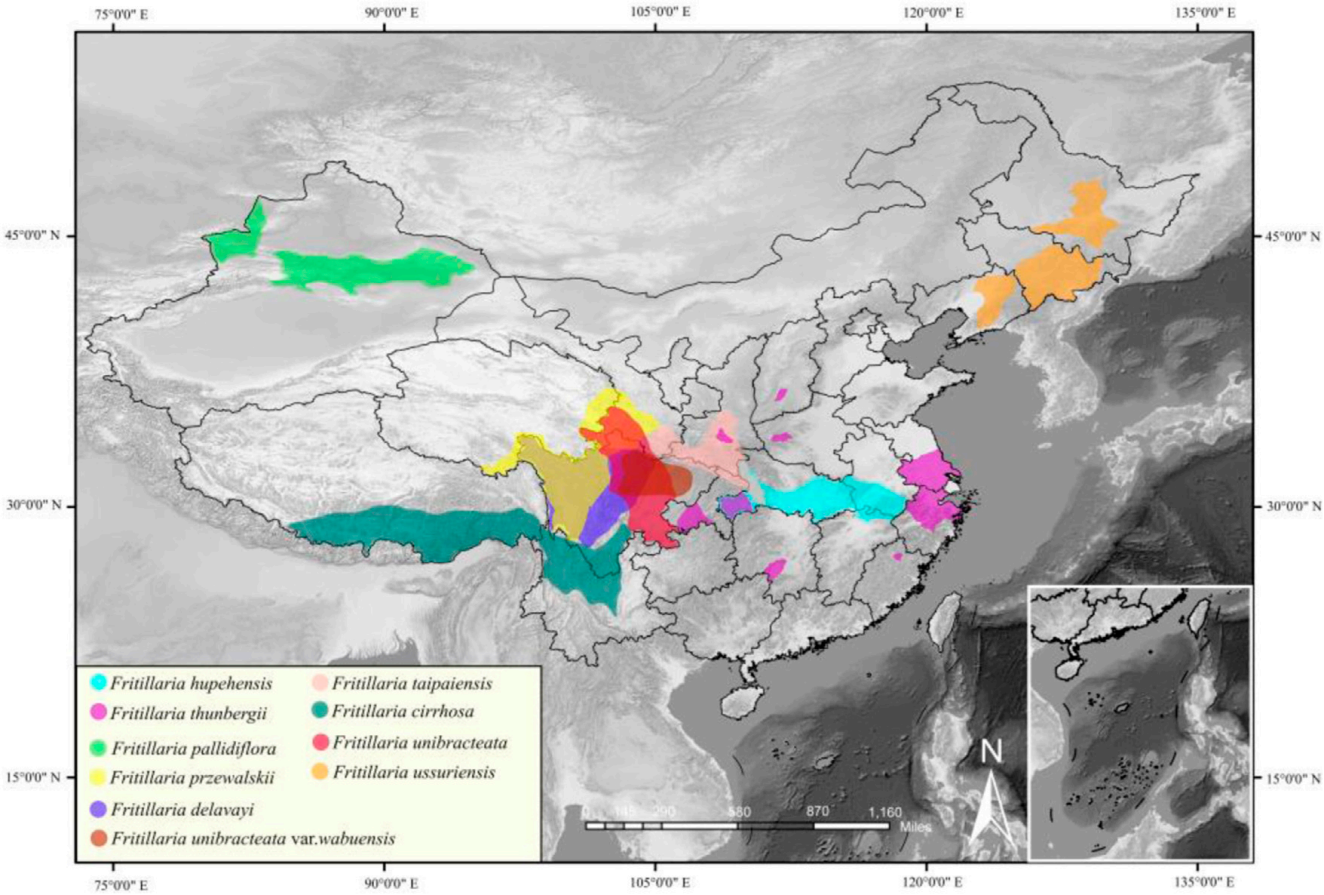
Common medicinal plants of the genus *Fritillaria* L. in China.

The medicinal quality and market price of *Fritillaria* L. from different places of origin have varied in recent years. To meet the requirements of the market, it is imperative to cultivate *Fritillaria* L. medicinal plants artificially. Five types of *Fritillaria* L. plants present in the Chinese Pharmacopoeia (Volume I, 2020 Edition) are collected; these include *Fritillaria ussuriensis* Maxim, *Fritillaria pallidiflora* Schrenk, *Fritillaria thunbergia* Miq., *Fritillaria hupehensis* Hsiao et K. C. Hsia, and *Fritillaria cirrhosa* D. Don. The specific growth environment and distribution of different *Fritillaria* medicinal plants are shown in Figure 1. The various *Fritillaria* medicinal plants have wide distribution range and high adaptability. It has been proven that it is feasible to breed wild *Fritillaria* medicinal plants (Huang et al., 2018; Luo M. et al., 2021a; Luo S. et al., 2021b). Five types of these *Fritillaria* plants all have the function of reducing heat, relieving coughs, reducing phlegm, and there are moistening lung function of *F. ussuriensis* and *F. pallidiflora,* detoxification, dissipating mass and eliminating carbuncle function of *F. thunbergia*, dissipating mass function of *Fritillaria hupehensis,* moistening lung, dissipating mass and eliminating carbuncle function of *F. cirrhosa* (Chinese Pharmacopoeia Commission, 2020).

## Diversity of rhizosphere microorganisms associated with medicinal *Fritillaria* plants

As the most active component of the soil ecosystem, rhizosphere microorganisms play an important role in plant growth, nutrient circulation, and improving yields (Mendes et al., 2013). The diversity and colonization ability of soil microbial communities in different microhabitats affect the growth rate of pathogens and also play an important role in improving plant health (Li et al., 2013). The abundance and diversity of soil microbial community decreased in the field evaluation of root rot disease in *Fritillaria ussuriensis*, while the population of pathogens in the healthy soil sample was quite low, indicating that the microbial community structure affected the health of *F. ussuriensis* (Song et al., 2016). The diversity and structure of the microbial community were closely related to the health level of *F. ussuriensis*, this can lay a foundation for the systematic study on the interaction between microbial genera in the pathological process of *F. ussuriensis* (Jiao et al., 2022).

### Diversity of bacteria in rhizosphere soil of *Fritillaria* medicinal plants

As the most abundant and widely distributed group of rhizosphere soil microorganisms, bacteria account for 70–90% of the total number of rhizosphere microorganisms, which are sensitive indicators of nutrient changes in rhizosphere soil (Yu et al., 2019). Researches about the relationship between the number and diversity of bacteria and *Fritillaria* medicinal plants were carried out. Liao (2011) found that the number of culturable bacteria of rhizosphere soil of *F. thunbergii* was decreased with the planting year increaced, which is related with the monocropping obstacle of *F. thunbergii.* In agricultural production, a variety of issues, such as disease accumulation, impaired growth, and quality decline, appeared in the third year after planting *Fritillaria taipaiensis* P. Y. Li seedlings (Mu et al., 2019a). This seriously affected the yield and quality of *F. taipaiensis*. The number of culturable rhizosphere microorganisms associated with wild and cultivated *F. taipaiensis* in different growth years and production areas was measured. It was found that the rhizosphere microorganisms of *F. taipaiensis* were abundant, and the number of bacteria was 2 × 10^6^ cfu·g^−1^–3×10^7^ cfu·g^−1^, when the total number of microorganisms was 3.8 × 10^7^ cfu·g^−1^, but which decreaced alongwith the planting years of cultivated *F. taipaiensis* and growth year of wild *F. taipaiensi* (Mu, 2019b).

Using the high-throughput sequencing technology, from the phylum level analysis, it can be seen that the bacterial community within the soil rhizosphere was mainly composed of Proteobacteria Acidobacteria, *Bacteroidetes*, *Verrucomicrobia*, *Firmicutes*, *Gemmatimonadetes*, *Actinobacteria*, *Planctomycetes*, *Nitrospirae*, and Chloroflexi (Mu, 2019). Among them, Proteobacteria belonged to the dominant phylum with the largest relative abundance (54.82%). As planting years increased, the relative abundances of the genera *Lactobacillus*, *Gemmatimonas*, *Bryobacter*, and *Bacteroides* gradually decreased, whereas the relative abundances of the genera *Methylotera*, *Sphingobacterium*, and *Pseudomonas* gradually increased (Mu, 2019). Wu (2021a,Wu 2021b) reported that the composition of phyla and genera within soil bacterial communities changed alongwith the growth years of *F. kansuensis*. Among them, for the relative abundance, Actinomycetes (23.58%–32.08%), Proteobacteria (22.00%–28.80%), Acidobacteria (13.82%–20.86%) and Chloroflexi (8.43%–15.08%) belonging to the dominant phyla, and the continuous planting of *Fritillaria kansuensis* for 5 years would significantly reduce the diversity of bacterial communities in the soil rhizosphere. Tang et al. (2021) discovered that the bacterial diversity in the rhizosphere gradually decreased as the growth of *Fritillaria thunbergii*.

The bacteria that presented decreased relative abundance in rhizosphere soil included potassium-solubilizing bacteria (PSB), phosphate-solubilizing bacteria, and nitrogen-fixing bacteria, which may because of the increase in the relative abundance of saprophytic fungi. Phosphate solubilizing bacteria can convert insoluble phosphorus into effective phosphorus that can be absorbed and subsequently used by plants. According to the different substrates of phosphate-solubilizing bacteria, they could be divided into organic phosphorus bacteria and inorganic phosphorus bacteria (Chi et al., 2021), which is a type of bacteria isolated from soil that can decompose phosphorus-containing minerals. PSB, also known as potassium bacteria or silicate bacteria, convert mineral potassium into potassium that is available for plant absorption and utilization by adhering to the mineral surface and releasing acidic substances. These are a type of bacteria isolated from the soil that can decompose potassium-containing minerals (Han et al., 2022).

The isolation and screening of organophosphate-solubilizing bacteria, inorganic phosphate-solubilizing bacteria, and PSB from the rhizosphere soil of *F. taipaiensis* in the previous report revealed that these varied with different growth years (Table 1). As the planting years of *F. taipaiensis* increased, there was an increase and subsequent decrease in the total number of organophosphate-solubilizing bacteria, Actinomycetes, inorganic phosphate-solubilizing bacteria, as well as other bacteria and microorganisms in the rhizosphere. However, there was a decrease in the number of PSB. It was found that fertilization and continuous cropping significantly affected the community composition and function of rhizosphere microorganisms associated with *F. taipaiensis*.

**TABLE 1:** Diversity of potassium solubilizing bacteria and phosphate solubilizing bacteria in the rhizosphere of *Fritillaria* L. medicinal plants.

Microbial strains	Numbering	Latin name	Similarity (%)	Reference strain registration sequence
Organophosphate solubilizing bacteria	YPB1	*Pseudomonas putida*	99	AY823622.1
	YPB2	*Pseudomonas lini*	100	DQ377769.1
	YPB4	*Pseudomonas salomonii*	99	JX840372.1
	YPB5	*Pseudomonas veronii*	99	JQ317807.1
	YPB6	*Pseudomonas mandelii*	100	DQ377771.1
	YPB7	*Pseudomonas fluorescens*	100	KJ420524.1
	YPB8	*Pseudomonas fluorescens*	100	KC018467.1
	YPB9	*Pseudomonas fluorescens*	100	KJ420524.1
	YPB11	*Pseudomonas fluorescens*	100	KJ420524.1
	YPB13	*Pseudomonas fluorescens*	100	KC018467.1
	YPB15	*Pseudomonas koreensis*	100	KF424274.1
	YPB17	*Pseudomonas poae*	100	JX515573.1
	YPB20	*Pseudomonas fluorescens*	100	KC018467.1
	YPB21	*Bacillus thuringiensis*	100	CP004858.1
	YPB22	*Pseudomonas grimontii*	100	KJ420529.1
	YPB24	*Pseudomonas migulae*	100	DQ377742.1
	YPB25	*Pseudomonas azotoformans*	100	KF040474.1
	YPB26	*Pseudomonas fluorescens*	99	KC018467.1
Phosphorus solubilizing bacteria	WP1	*Pseudomonas fluorescens*	99	KJ756336.1
	WP4	*Arthrobacter nicotinovorans*	100	KR922212.1
	WP5	*Arthrobacter nicotinovorans*	100	KR922212.1
	WP10	*Phyllobacterium myrsinacearum*	100	KJ147062.1
	WP11	*Arthrobacter ilicis*	99	KR088423.1
	WP13	*Phyllobacterium myrsinacearum*	100	KJ147062.1
	WP18	*Phyllobacterium myrsinacearum*	100	KJ147062.1
Potassium solubilizer	GB1	*Pseudomonas fluorescens*	100	HQ606463.1
	GB13	*Agrobacterium tumefaciens*	100	CP011247.1
	GB15	*Agrobacterium tumefaciens*	100	CP011247.1
	GB23	*Pseudomonas fluorescens*	100	HQ606463.1
	GB25	*Agrobacterium tumefaciens*	99	CP011247.1
	GB33	*Agrobacterium tumefaciens*	100	CP011247.1

### Diversity of fungi in the rhizosphere of *Fritillaria* medicinal plants

It was discovered that the symbiosis between *F. Taipaiensis* and AM fungi was very common; different AM fungi infected the root system of *F. Taipaiensis* to different degrees; and the inoculation of AM fungi significantly increased the mycorrhizal infestation rate, and, in turn, the biomass of *F. taipaiensis* (Zhang, 2020). AM fungi could increase the content of inorganic elements in the rhizome and soil of *Fritillaria taipaiensis* P. Y. Li.; enhance the enrichment ability of inorganic elements, thus improving the growth and development of *F. taipaiensis* P. Y. Li.; and promote the accumulation of nutrients in medicinal material (Wei et al., 2021). *Fritillaria thunbergii* Miq. is a crop that is continually cropped; thus, it is necessary to change the land after 1 year of artificial planting (Yu and Wu, 2017). When the four planting years increased, there was a linear increase in the number of cultivable fungi in the soil from the root zone of *F. thunbergii* Miq., whereas the total amount of cultivable Actinomycetes, bacteria decreased linearly (Liao et al., 2011). In general, fungi prefer acidic environments, Actinomycetes prefer neutral to alkaline environments, while other bacteria mainly live in neutral environments. The continuous cropping of *F. thunbergii* Miq. reduced the pH value of the soil, resulting in the soil being slightly acidic. This, being conducive to the propagation of fungi, results in the transition of the soil from a high fertility “bacterial type” to a low fertility “fungal type” (Liao et al., 2011). As the planting years of *F. taipaiensis* increased, so did the fungi in the rhizosphere. This indicates that long-term continuous cropping would lead to a reduction in the numbers of beneficial microorganisms, and increase the numbers of pathogenic microorganisms in the rhizosphere of *F. taipaiensis* (Mu et al., 2019b; Gu et al., 2020), which is not conducive to the growth of *F. taipaiensis*.

Non-culture methods can more accurately reflect the community structure of rhizosphere microorganisms compared with traditional culture methods. The results of high-throughput sequencing of ITS sequence showed that fungal community in the rhizosphere of *F. taipaiensis* was mainly composed of Ascomycota, Zygomycota, Basidiomycota, Glomeromycota, Neocallistigomycota, and Chytridiomycota. And Ascomycota fungi were the most dominant population. With the increase in planting years, the relative abundance of *Pseudogymnomyces* in the soil rhizosphere gradually decreased, along with the relative abundance of populations such as pathogenic fungi in *Fusarium*, *Gibberella*, *Rhizopus*, *Colletotrichum*, and *Peziza* (Mu, 2019; Zhou et al., 2021).

## Interaction between *Fritillaria* plants and rhizosphere microorganisms

### Effects of root exudates of *Fritillaria* medicinal plants on rhizosphere microorganisms

The richness and diversity of rhizosphere microorganisms plays a key role in regulating ecological functions, such as organic matter decomposition, nutrient cycling, and soil carbon dynamics (Sasse et al., 2018). In nature, plants release and secrete various compounds into the environment through a variety of ways, such as *via* leaf litter, root residues, and root exudates. This occurrence provides vital carbon sources for the formation of aggregates, and affects the physical and chemical properties of community of rhizosphere microorganisms (Vezzani et al., 2018; Vives-Peris et al., 2020). Conversely, the biological, physical, and chemical characteristics of rhizosphere microorganisms affects host plants and their coexisting plants (Ehrenfeld et al., 2005). Root exudates are important factors in the formation of rhizosphere microorganisms, because the various primary metabolites and secondary metabolites secreted by roots can play the role of shaping, interfering, or transmitting signals to change the rhizosphere microflora, recruit and promote beneficial microorganisms, and resist harmful microorganisms (Venturi and Keel, 2016). Generally, the rhizosphere microorganism of plant are more abundance, active and rich in diversity than the no-rhizosphere microorganism.

Root exudates provided abundant nutrients and energy for the growth of rhizosphere microorganisms, and also affects the distribution, species, and quantity of rhizosphere microorganisms (Wang et al., 2007). The root exudates of *Fritillaria* medicinal plants include organic acids, amino acids, soluble sugars, total phenolic acids, and organic substances (Guo et al., 2013). Various organic substances, such as ethers, olefins, acids, aldehydes and ketones, esters, alkanes, ureas, phenols, and alcohols (Wang et al., 2009) have been found in root exudates. Among them, are high contents of aldehydes and ketones. Phenolic acids secreted by roots promote the growth of black spot fungus and *Botrytis cinerea* (Guo et al., 2013). Phenol and 1,3,5-triallyl-1,3,5-triazine-2,4,6 (1H, 3h, 5H) trione, being the main root exudates of *Fritillaria*, had a significant inhibitory effect on the growth of its seedlings (Wang, 2010; Wang et al., 2010a,b). Generally, the secondary metabolites produced by the roots of medicinal plants? were easily released into the soil, thereby promoting the colonization and shaping of the rhizosphere microorganism community, and driving the feedback effect of plant soil on defense and growth (Hu et al., 2018). This results in changes in the population structure of plant rhizosphere microorganisms. It should be noted that there are great differences in the species, dominant species and quantity of rhizosphere microorganisms in different growth stages of the same plant or in the same growth stage of different plants. This is due to the variety and quantity of plant root exudates, which vary with growth stage or plant variety (Brimecombe et al., 2001).

### Effects of rhizosphere microorganisms on the growth and effective components of *Fritillaria* medicinal plants

The metabolism of rhizosphere microorganisms either directly promotes or inhibits the nutrient absorption and growth of roots, which plays a key role in the secondary metabolism of plants. Therefore, in recent years, much attention has been paid to the interpretation of the quality formation, change, and mechanism of traditional Chinese medicine from the perspective of microecology, to clarify the influence of rhizosphere microorganisms on the growth of medicinal plants and medicinal ingredients. It has been confirmed that rhizosphere microorganisms improve the yield and medicinal components of medicinal plants. Zhang et al. (2020) inoculated four types of arbuscular mycorrhizal fungi (AMF) including *Glomus constrictum* (GC), *Glomus versiforme* (GV), *Glomus mossae* (GM), and *Glomus aggregatum* (GA). AMF alone could significantly increase the fresh weight, dry weight, and drying rate of *F. taipaiensis* bulbs, and increase the contents of Fritillarin A, sibelline glycoside, Fritillarin B, Fritillarin, and alkaloids in the bulb. AM fungi could increase the content of inorganic elements in the rhizome and soil of *F. ussuriensis*, and also enhance the enrichment ability of inorganic elements, thereby improving the growth and development of *F. ussuriensis*, and promoting the accumulation of nutrients in medicinal materials (Wei et al., 2021). Sun et al. (2022a) found that medicinal plants had a great diversity of rhizosphere AM fungi. Fungal colonization improved plant growth performance and root morphology, and significantly increased the content of most disaccharides, but either reduced or did not change the content of most monosaccharides. AM fungi significantly increased the concentration of the medicinal components (chrysophanol, physion, polydatin, and resveratrol) in the root of *P. cuspidatum*, and upregulated the expression of related synthase genes (Sun et al., 2022b). Therefore, AM fungi are beneficial to the growth and nutrient absorption of medicinal plants, thereby accelerating the accumulation of medicinal ingredients.

Pan et al. (2010a) used *Bacillus subtilis* to spray leaves and irrigate roots at different growth stages of *F. pallidiflora*, which increased the yield by 16.8%. The correlation analysis between rhizosphere microorganisms and the content of Siberian in the growth stage of *F. pallidiflora* showed that the fungi in rhizosphere soil had a significant positive correlation with the content of Siberian, while Actinomycetes and bacteria had a positive correlation with the content of Siberian (Pan et al., 2010b). These results are similar with those reported by Qiu (2010), and have the potential to be included in applications to further develop the resources of beneficial fungi in the rhizosphere of *F. pallidiflora* and improve its medicinal quality. Zhao et al. (2021) isolated 20 endophytic bacteria from 3-year-old *F. przewalskii* Maxim. plants, which were distributed in the three phyla of Proteobacteria, Firmicutes, and Actinomycetes. Among them, strains related to *Bacillus, Rhizobium*, and *Pseudomonas* were the dominant growth-promoting bacteria. Foliar spraying of the Firmicutes *Bacillus*, *Rhizobium*, and *Pseudomonas* compound promoted the growth of *F. przewalskii* Maxim. and significantly increased its yield. Tang et al. (2021) found that with the advancement of the growth process of *F. thunbergii* Miq., the content of alkaloids (Fritillarin A and Fritillarin B) in the bulb first increased and then decreased. Correlation analysis showed that the Chao1 index and Shannon index among the bacteria were positively correlated with the content of monomer alkaloids and total alkaloids, indicating that the soil bacterial community was closely related to the content of alkaloids. Furthermore, the secondary metabolites produced by *Fritillaria cirrhosa* D. Don displayed high antioxidant activity. For example, the low polar substances extracted by petroleum ether had weak ABTS free radical scavenging activity, whereas the high polar substances extracted by solvents such as n-butanol and ethyl acetate had high ABTS free radical scavenging activity (Pan et al., 2017). These results indicated that it was feasible to use beneficial rhizosphere microorganisms to improve the yield and quality of *F. taipaiensis* in artificial cultivation. During the long-term artificial cultivation of medicinal plants, agricultural management measures (Tan et al., 2013; Geng et al., 2018), such as the chemical fertilizer, pesticide spraying, and planting method caused a change in the rhizosphere microbial biomass and its community structure, which was mediated by soil physical and chemical properties. This resulted in varying degrees of differences in the medicinal value between wild medicinal materials and cultivated medicinal materials, which further increased the variation in quality of the same type of Chinese medicinal materials from different origins (Schmidt et al., 2019).

Microbial fertilizers are a type of live microbial preparation widely used in agricultural production. These play an irreplaceable role in future advancements in agriculture due to their contributions to environmental protection, soil improvement, fertility improvement, output increase, and quality improvement. For example, the exogenous inoculation of AMF, phosphorus-dissolving bacteria, potassium-dissolving bacteria, and other microbial fertilizers increased the alkaloid content of 3-year-old *F. taipaiensis* plants (Ma, 2021). Based on the results of the content of secondary metabolites, and relevant physical and chemical properties of rhizosphere soil, the quality of *F. taipaiensis* had a great relationship with rhizosphere microorganisms. However, the growth potential of seedlings co-cultured with a single strain was relatively low, and some even exhibited yellowing. After mixed inoculation of AMF, phosphorus-dissolving bacteria, and potassium-dissolving bacteria, the alkaloid content in bulbs was lower than that in *F. taipaiensis* plants that were co-cultured with single strains. This may be because of the limitation of a single strain, the unstable interaction between rhizosphere promoters and host plants (Rainer and Florian, 2018), or it may be due to the effect of unknown ecological functions (Lebeis et al., 2012). Therefore, the rhizosphere microorganisms of plants may not exist alone, but instead play a role in the active ingredients of *F. taipaiensis* in the form of a specific flora. In addition, the exogenous application of microbial agents increased the number of Actinomycetes and bacteria in the rhizosphere soil of 2-year-old *F. taipaiensis* plants, but had no obvious effect on the number of fungi. It has also significantly improved the activities of soil protease, acid phosphatase, urease, and sucrase, thereby playing an important role in the sustainable utilization and stability of soil nutrients (Dong, 2018). The exogenous application of different doses of microbial fertilizer significantly increased the height of the plant, the fresh weight of the plant and bulb, and the yield of *F. pallidiflora*. Therefore, microbial fertilizer was commonly applied in the cultivation of *F. pallidiflora*. It was more beneficial to increase the production and efficiency of *F. pallidiflora* by opening ditches and applying the microbial fertilizer during the growth period (Chen et al., 2017). Therefore, microbial fertilizer can improve the structure and density of the microbial population in the rhizosphere soil, as well as the quality.

### Hazards of rhizosphere microorganisms on *Fritillaria* medicinal plants

*Fritillaria* Chinese herbal medicines generally take 4–6 years to grown from a germinating seed to flowering and bearing, and take at least 3 years for commercial medicinal materials. However, with the increase in the growth years of *Fritillaria* Chinese herbal medicines, conditions that are favorable for outbreaks of harmful rhizosphere microorganisms have been created in the process of artificial planting. Coupled with poor field management, the quality of medicinal materials gradually decreased. Common soil borne diseases, such as root rot disease, sclerotium disease, ray mold disease, rust disease, and yellow rot rust, occurred frequently and caused serious harm (Wang, 2010). This made it very easy for *Fritillaria* to develop bulb rot and lose its medicinal value (Liu et al., 2020; Wu, 2021a). Using morphological identification method, the pathogen of sclerotium disease and root rot disease of *F. pallidiflora* was identified as *Stromatinia rapulum* (Supi et al., 2012) and *Fusarium solani* var. *coeruleum* (Supi et al., 2015), respectively. But *Sclerotium denigrans* and *Sclerotinia sclerotiorum* caused the sclerotium disease of *F. ussuriensis* with molecular biology methods (Song et al., 2016a). The pathogens of root rot disease in *F. thunbergia* were *Fusarium* oxysporum and *F. incarnatum*, the pathogen of black spot disease was *Alternaria alternata*, and *Phoma* sp. could cause leaf spot disease (Wang, 2017). But the pathogens of root rot disease of *F. przewalskii* were *F. oxysporum*, *F, tricinctum*, *Bionectria ochroleuca* and *Clonostachys rosea* (Wu et al., 2021a). *Botrytis cinerea* was identified as the pathogen of ray mold disease of *F. thunbergia,* and not the *Botrytis elliptica* which was generally considered as the pathogen in the past (Li et al., 2022).

## Application of rhizosphere microbiome in the cultivation of *Fritillaria* medicinal plants

The plant microbiome, also known as the pan-genome, refers to the study of the role of the rhizosphere microbial community in the growth of host plants in a holistic manner. It plays an irreplaceable role in the ecological environment and agricultural production (Bulgarelli et al., 2013; Andezej and Philip, 2015), which is recognized as an important determinant of healthy plant growth (Berg et al., 2014). Combining the core strategy of “whole microbiome association analysis,” the microbiome accurately decoded the expression spectrum, composition spectrum, and function spectrum of the community/flora, discovered key organisms and their markers, and then clarified the complex causal chain and interaction mechanism between the “microbiome plant soil” (Claire, 2017).

### Classification of rhizosphere microbiome

According to the varied distribution of microorganisms on the root system, the rhizosphere microbiome could be divided into the three categories of rhizosphere microbiome, root surface microbiome, and root internal microbiome (Tripathi et al., 2018). Due to the limitations of research technology, these three aspects had been involved in previous studies, but they were not specifically analyzed as a whole to evaluate the importance of these rhizosphere microbiomes to plant growth. In the study of plant rhizosphere microbiome, it is necessary to pay attention to the different sampling methods of different microbiomes. After the plant roots were taken out of the soil, 1 mm-thick soil was collected from around the roots by shaking and washing. In this way, all the soil on the root surface was placed in a phosphate buffer solution, and the microorganisms close to the root surface were separated for rhizosphere microbiome analysis (Edwards et al., 2015). Because there were few microorganisms on the root surface and it was difficult to collect them, the cleaned roots were put into phosphate buffer solution for ultrasonic treatment for 30 s, and the small amount of plant soil residues in the buffer solution were considered the microbial components on the root surface. After the cleaned roots were chemically treated with sodium hypochlorite and ethanol, or all the microorganisms on the root surface were removed by continuous sonication performed twice, the roots were then crushed by adding glass beads, which was considered the microbial components in the roots (Lundberg et al., 2012; Reinhold-Hurek et al., 2015).

### Study on rhizosphere microbiome in the cultivation of *Fritillaria* medicinal plants

A large number of studies have indicated that the rhizosphere microbiome played an important role in promoting the growth and tolerance of host plants. These are mainly divided into two methods of negative interaction such as rhizosphere microorganisms infecting the host to cause diseases or competing with the host for nutrition, and positive interaction such as rhizosphere microorganisms promoting growth, stress resistance, and disease resistance (Bais et al., 2006). This interaction process exerts a strong selection pressure on both the rhizosphere microbiome and host plants, thus forming the mode and rate of rhizosphere microbial evolution and affecting the formation of the rhizosphere microbiome (Cosetta and Wolfe, 2019). An in-depth exploration of the selection mechanism of plants for the rhizosphere microbiome could guide the recombination and improvement of the rhizosphere microbiome in practical applications (Sun et al., 2021). It also improved the understanding of plant microbe interaction in theory, which is of great significance for future agricultural production. For example, using Indica rice and Japonica rice as experimental materials, Zhang et al. (2019) found that Indica rice often showed higher nitrogen utilization efficiency than that of Japonica rice. Furthermore, the diversity of the rhizosphere microbiome of Indica rice was significantly higher than that of Japonica rice, which directly confirmed the relationship between the rhizosphere microbiome and the nitrogen utilization efficiency of plants. Utilizing beneficial members of the rhizosphere microbiome and making microbial fertilizer is a very promising application within fertilizer preparation (Hu et al., 2016). Unfortunately, the current research on the development of the rhizosphere microbiome into microbial fertilizer is still at the experimental stage. Nevertheless, microbial fertilizers have potential use in the natural agricultural environment.

Yuan et al. (2018) found that when plants were infected by pathogenic bacteria, they could recruit and enrich certain beneficial microorganisms by sending specific signals. This phenomenon was the famous “cry for help hypothesis.” These beneficial recruited microorganisms in the rhizosphere improved the disease resistance of plants in four ways: competition, parasitism, antibiosis, and the induction of systemic resistance (Mendes et al., 2013). For example, the antibacterial metabolite 2,4-diacetyl-phloroglucinol secreted by *Pseudomonas fluorescens* in rhizosphere microorganisms inhibited *Sclerotium rolfsii* by more than 75% (Asadhi et al., 2013). *P. fluorescens* (WCS417r) that colonized the Arabidopsis rhizosphere could upregulate the expression level of defense-related genes of the host plant pathogen (*Pseudomonas syringae* pv *tomato*) (Wees et al., 1999). In addition, AMF established a good symbiotic system with most terrestrial plants, promoted its external hyphal network to accelerate the absorption of nutrients and water, and enhanced the resistance of host plants (Li et al., 2019). It could be seen that no matter how the rhizosphere microorganisms interact with plants, the successful colonization of microorganisms in the rhizosphere was of great importance to plants. This is because the different secretions secreted by plant roots were signals recognized by rhizosphere microorganisms, and also vectors for mutual communication between rhizosphere microorganisms. Therefore, these signals could, in-turn, be used for the colonization of rhizosphere microorganisms. Consequently, root exudates are considered to be important mediators in the communication between rhizosphere microorganisms and host plants, due to the diversity and complexity of their components. Currently, there is little research on the rhizosphere microbiome of *Fritillaria* plants; thus, the research on the rhizosphere microbiome will facilitate the recruitment of beneficial bacteria, and facilitate the development of *Fritillaria* resources.

## Conclusion and future perspectives

In this review article, rhizosphere microorganisms were found to be closely related to the propagation, metabolism, and growth of *Fritillaria* medicinal plants. Therefore, the rhizosphere microorganisms of *Fritillaria* plants have received much attention, which not only helps to solve the practical problems of disease prevention among *Fritillaria* medicinal plants, but also improves the yield and quality of these plants. It also helps to clarify the interaction mechanism between *Fritillaria* medicinal plants and beneficial or harmful microorganisms in the rhizosphere. Based on the authors’ knowledge, future research on rhizosphere microorganisms of *Fritillaria* medicinal plants should focus on the following aspects.

### Optimizing research methods for rhizosphere microbial diversity among *Fritillaria* medicinal plants

Presently, the traditional method used on rhizosphere microorganisms is the pure culture method. With the development of molecular biology technology, the free culture method is widely used in the research of rhizosphere microorganisms of medicinal plants. However, the free culture method cannot obtain live strains, nor provide strains for the research of growth-promoting bacteria in the rhizosphere. Therefore, in the research of rhizosphere microbial diversity of *Fritillaria*, it is suggested to combine the pure culture method and free culture method. Moreover, there are few reports on the diversity of the rhizosphere microbial population of *Fritillaria* medicinal plants. Because most of the reports are only at the pure culture stage, in the future more attention should be carried out to explore the impact of different *Fritillaria* medicinal plant varieties, growth years, environmental conditions, and climate factors on the rhizosphere microbial diversity of these plants. The relationship between the rhizosphere microorganisms of *Fritillaria* medicinal plants and their growth should be understood in detail.

### Improving the understanding of the relationship between medicinal plants of *Fritillaria* and rhizosphere microorganisms

The mechanism of interaction between *Fritillaria* medicinal plants and rhizosphere microorganisms is not clearly understood. In the future, more attention should be paid to the growth-promoting mechanism and the mechanism of the continuous cropping obstacle of *Fritillaria* medicinal plants. Moreover, the development of biocontrol agents should be strengthened. The perennial *Fritillaria* medicinal plants have suffered from widespread and serious underground diseases and insect pests, resulting in a decline in the quality of *Fritillaria* Chinese medicinal materials. Therefore, microbial agents that are safe, efficient, and pollution-free should be developed, which can improve the soil environment, and the output and quality of *Fritillaria* medicinal plants (Dong, 2018).

### Revealing the relationship between diseases of *Fritillaria* medicinal plants and rhizosphere microorganisms

At present, pathogens of main diseases of *Fritillary* medicinal plants have been reported (Supi et al., 2012; Supi et al., 2015; Song et al., 2016a; Wang, 2017; Wu et al., 2021; Li et al., 2022), but some pathogens are only identified by morphological identification method, and need to be further identified by molecular biology method. The pathogen of a certain plant of *Fritillaria* may be speculated from the incidence of other *Fritillaria* plants, but the pathogen of the same disease in different *Fritillaria* plants are not exactly the same. Therefore, it is necessary to strengthen the research on the pathogenic microorganisms of diseases and insect pests of *Fritillaria.* Moreover, it is necessary to strengthen the research on rhizosphere microorganisms of diseased plants and healthy plants, explore which changes of rhizosphere microorganisms are *related* to the occurrence of medicinal plant diseases of *Fritillaria*, and screen biocontrol strains from rhizosphere microorganisms. Moreover, it is necessary to strengthen the research on rhizosphere microorganisms of diseased plants and healthy plants, explore which changes of rhizosphere microorganisms are related to the occurrence of medicinal plant diseases of *Fritillaria*, and screen biocontrol strains from rhizosphere microorganisms.

Currently, it would be more meaningful to develop more field applications for rhizospheric microbes rather than potted plants. The research methods on the rhizosphere microorganisms of *Fritillaria* medicinal plants are relatively isolated, thus it is necessary to learn from the research methods between other medicinal plants and rhizosphere microorganisms to understand the interaction mechanism between *Fritillaria* medicinal plants and rhizosphere microorganisms. In summary, it is expected that this review article will facilitate the development of *Fritillaria* medicinal plants.
